# Mode of Action of Zn-DOPOx and Melamine Polyphosphate as Flame Retardants in Glass Fiber-Reinforced Polyamide 66

**DOI:** 10.3390/polym14183709

**Published:** 2022-09-06

**Authors:** Johannes Klitsch, Rudolf Pfaendner, Claudia Fasel, Frank Schönberger

**Affiliations:** 1 Division Plastics, Fraunhofer Institute for Structural Durability and System Reliability LBF, 64289 Darmstadt, Germany; 2 Department of Material Science, Technical University Darmstadt, 64287 Darmstadt, Germany

**Keywords:** flame retardant, PA 66, phosphorus-containing flame retardant, metal phosphorous salt, halogen-free

## Abstract

In this study, the flame retardant effect of the Zn salt of 10-hydroxy-9,10-dihydro-9-oxa-10-phosphaphenanthrene-10-oxide (Zn-DOPOx), melamine polyphosphate (MPP) and their mixture was investigated towards the mode of action in glass fiber-reinforced polyamide 66 (PA 66 GF). The flammability was evaluated using UL 94 V and cone calorimetry. Influence on char formation was analyzed by SEM. Thermal decomposition of Zn-DOPOx and MPP was studied by TGA and ATR-FTIR. The release of gaseous PA 66 decomposition products was investigated using TGA-DTA-FTIR. Combining Zn-DOPOx and MPP leads to an improvement in flame retardancy, most pronounced for equal parts of weight. Mode of action changes significantly for Zn-DOPOx:MPP (1:1) compared to the sole components and a strong interaction between Zn-DOPOx and MPP is revealed, resulting in a more open char structure. Fuel dilution as well as less exothermic decomposition are essential for the mode of action of the combination. Through low HRR values and high CO/CO_2_ ratio during cone calorimetry measurements, a significant increase in gas phase activity was proven. Therefore, it is concluded that Zn-DOPOx:MPP (1:1) leads to a significant increase in flame retardancy through a combination of mode of actions in the gas and condensed phase resulting from the change in thermal stability.

## 1. Introduction

Engineering plastics, such as polyamides (PA) and polybutyleneterephthalate (PBT), are commonly used for a wide range of applications due to their versatile properties, e.g., good mechanical strength and chemical stability [1]. Despite the various advantages of polymeric materials, most of them exhibit the major drawback of intrinsic flammability due to their chemical composition [2,3]. For many applications (e.g., in the automotive sector and for electro and electronic) polyamide 66 (PA 66) is often reinforced with glass fibers in order to improve mechanical properties such as tensile strength and dimension stability. On the other hand, incorporation of glass fibers leads to an increase in heat conduction and higher flammability due to the wick effect similar to a candle [4]. 

Depending on the flame retardants, the effect of flame retardancy can be achieved through different modes of action taking place in the solid phase of the polymer or in the gaseous phase [5]. For PA 66, the use of halogenated compounds such as decabromodiphenyl ether (decaBDE) has proven to act as effective flame retardants [6]. These compounds interrupt the radical chain reaction in the gas phase. A major drawback of halogenated flame retardants is the release of toxic gases on decomposition. They are also toxic towards the environment and humans [6,7,8,9,10,11]. Therefore, regulatory actions are being implemented in some parts of the world, for example Europe (REACH), to minimize or even phase-out the use of halogen-containing flame retardants [12,13]. Alternative modern fire safety solutions are often based on the concept of using phosphorus-based flame retardants due to their various mode of action. Phosphorous species in higher oxidation states such as phosphates are more likely to their exhibit mode of action in the solid phase. Species in lower oxidation states, such as phosphinates, tend to act in the gas phase as radical generators to poison the flame [14,15,16,17]. Mode of action in the gas phase can be achieved through radical quenching and dilution of the combustible decomposition products. Solid phase mechanisms are often based on heat removal upon endothermic decomposition or char formation. Efficient flame retardancy is often realized by balancing the mode of action between the solid and gas phase.

**Scheme 1:**
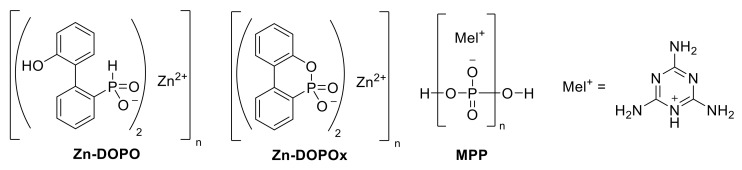
Chemical structures of Zn-DOPO, Zn-DOPOx and melamine polyphosphate (MPP).

Flame retardants for PA 66 need to be chemically stable at temperatures well above the processing temperature (T ≥ 280 °C). Phosphorus-containing salts are often used because of their high thermal stability. For glass fiber-reinforced PA 66 (PA 66 GF), the use of the aluminum salt of diethylphosphinic acid (DEPAL) proved to be very effective due to its high gas phase activity. A loading of ≥20% is needed in order to achieve good flame retardancy with V-0 classification in UL-94 V (0.8 mm) [18]. The required amount of DEPAL can be reduced by using melamine polyphosphate (MPP) as a synergist [19]. In addition, MPP shows a pronounced effect on char formation in PA 66 [20]. For the use of DEPAL and the combination with MPP, significant corrosion of the polymer melt on the processing machinery is reported in the patent literature [21]. The addition of small amounts of certain metal salts improved flame retardancy and reduced corrosion [22]. Zinc borate increases the yield of char and char stability due to the formation of more stable boron aluminum phosphates instead of aluminum phosphates when using only DEPAL and MPP [23]. Bourbigot et al. reported the use of metal-containing flame retardants by incorporating metal ions into the triazine-based polyphosphates structure via intercalation [24,25,26]. Compared to MPP, higher flame retardant efficiency was achieved, and by variation of the metal atom, the possibility to tune the flame retardant properties with regard to their thermal stability, charring ability, and influence on polymer decomposition was shown. In the field of phosphorus-based flame retardants, 10-hydroxy-9,10-dihydro-9-oxa-10-phosphaphenanthrene-10-oxide (DOPO) is known for its gas phase efficiency through decomposing into dibenzofurane while releasing phosphorous species (PO-radical and HPO) [27,28,29]. Therefore, the DOPO moiety is used in many syntheses as a building block to achieve gas phase activity [30,31,32,33,34,35,36,37]. While most organic DOPO-containing flame retardants are thermally not stable enough to be used in PA 66, some inorganic analogous have thermal stabilities >300 °C [38]. Therefore, the Zn salt based on the phosphonate analogue DOPOx structure will be investigated in the scope of the present paper. The DOPOx structure has been selected as the phosphinate analogue Zn-DOPO already exhibits a mass loss below the extrusion temperature of PA 66. The different chemical structures of the phosphinate salt (Zn-DOPO) and phosphonate salt (Zn-DOPOx) are reported in the literature [38] and are shown in Scheme 1.

The synthesis of metal salts based on DOPO/DOPOx was first described in 1977 [39]. Regarding the use of these salts as flame retardants in PA 66 GF, only one patent by Wehner [40] gives an example for the flame retardant performance of Zn-DOPO in combination with MPP and various metal triazinepolyphosphates which resulted in a V-0 classification (1.6 mm) at a total loading of 22.5%. In this patent, the phosphinate derivate Zn-DOPO was used. Compared to Zn-DOPOx, an additional decomposition step (T = 190−220 °C) could be observed [38]. The first mass loss can be assigned to the oxidative cyclisation to the phosphonate variant Zn-DOPOx [38,41]. Through this cyclization, water is released below the extrusion temperature of PA 66. Another patent published mentioning Zn-DOPOx describes the use of DOPO(x)-salts in combination with N-containing bases as ligand, however, only examples as single flame retardant in PA 6 were given [42]. Most recently, the use of mixed metal salts with DOPO(x) and hydroxyl-containing ligands has been described in the patent literature [43]. Although Zn-DOPOx is mentioned in the patent literature several times, the mode of action has not been further investigated so far. 

Following the known synergistic principle of MPP/DEPAL [23,44], coformulations of Zn-DOPOx with MPP are investigated towards the mode of action in glass fiber-reinforced PA 66. In order to evaluate the mode of action, different formulations in PA 66 GF using the single flame retardants and the binary mixture will be prepared and analyzed towards the fire behavior (UL-94 V and cone calorimetry). The protective layer (char) formed during fire testing will be evaluated with SEM towards the condensed phase activity. Thermal stability of the flame retardants and the influence on the thermal stability of the compounds will be determined using TGA. To further analyze the decomposition of the flame retardants and the interaction for Zn-DOPOx:MPP (w:w = 1:1), TGA residues prepared under nitrogen will be analyzed with ATR-FTIR spectroscopy. For the compounds additional information regarding the gas phase (release of inert and combustible C−H fragments) and condensed phase (exothermic and endothermic decomposition behavior) contributions are accessed through DTA-FTIR analysis.

## 2. Materials and Methods

### 2.1. Compounding and Preparation of Test Specimen

PA 66 GF samples (30% GF) containing Zn-DOPOx and MPP were prepared with a total loading of 22, 20, and 18 wt. % according to Table 1.

The materials used were PA 66 (Ultramid^®^ A3K, BASF SE, Ludwigshafen, Germany), glass fibers (CS7928, Lanxess Deutschland GmbH, Köln, Germany) with a diameter of 11 µm (nom.) and an average fiber length of 4.5 mm (nom.), calcium stearate (Ceasit AV, Baerlocher GmbH, Unterschleißheim, Germany), Zn DOPOx (DOPO Zink Polymer S20, Metadynea Austria GmbH, Krems, Austria), MPP (Budit® 342, Chemische Fabrik Budenheim KG, Budenheim, Germany). The compounds were prepared using a 27 mm twin screw extruder ZSE 27 (Leistritz, Nuremberg, Germany) with a process temperature of 280 °C except for the nozzle, which was heated to 300 °C. The rotational speed of the screws was 300 rpm with a throughput of 5 kg/h. PA 66 was dried at 80 °C before processing until humidity was below <0.06%. Zn-DOPOx and MPP were dried under vacuum at 80 °C for at least 4 h before compounding. For better processability, 0.2% calcium stearate was used as a lubricant. The specimens used for UL-94 V and cone calorimetry were prepared using an injection molding machine 320A (Arburg, Loßburg, Germany) with a plastification temperature of 295 °C and a molding tool temperature of 95 °C.

### 2.2. Thermal Analysis

Thermogravimetric analysis (TGA) was performed using the device TGA 2 (Mettler Toledo, Columbus, OH, United States of America). All experiments were carried out in 150 µL alumina pans. Nitrogen or air flow of 50 mLmin^−1^ and a heating rate of 10 Kmin^−^^1^ were applied. The temperature range was between 30 °C and 900 °C. Derivative thermogravimetry (DTG) was used to visualize and compare the mass loss based on the thermograms.

### 2.3. Fire Testing

Compounds were tested regarding their flame retardancy performance using the UL 94 V method according to IEC 60695-11-10 on a UL-94 testing device (Dr. Ing. Georg Wazau Mess- und Prüfsysteme GmbH, Berlin, Germany). The testing was carried out using the standard specimen dimensions of 125 mm length × 12.5 mm width at thicknesses of 0.8 mm and 1.6 mm.

Cone calorimeter (Dr. Ing. Georg Wazau Mess- und Prüfsysteme GmbH, Berlin, Germany) experiments were performed based on ISO-5660-1 [45]. The only exception from the standard procedure was the fixed measurement duration of 900 s for all compounds. The specimen dimensions were 100 mm length × 100 mm width × 3.2 mm thickness. The samples were tested using a metal frame sample holder. An external heat flux of 50 kWm^−2^ was applied.

### 2.4. Residues Analysis

Analysis of different types of residues was made using ATR-FTIR spectroscopy, SEM, or SEM-EDX regarding the elements C, N, O, P, and Zn. ATR-FTIR spectroscopy was done using the Nicolet type Nexus 670 (Thermo Fisher Scientific, Waltham, MA, United States of America) with an ATR Golden Gate module. The samples used in ATR-FTIR analysis were prepared in TGA-experiments in which they were cooled down with a cooling rate of 100 Kmin^−1^ under nitrogen after reaching the target temperature.

The SEM analysis of burnt specimens was carried out on a scanning electron microscope SM300 (Topcon Corporation, Tokyo, Japan) with an acceleration voltage of 20 kV.

SEM-EDX was performed on the scanning electron microscope JSM-7600F (Jeol, Akishima, Japan) with the sensor X-Max-80 mm^2^ (Oxford Instruments, Abingdon, United Kindom) with an acceleration voltage of 15 kV, 114.6 µA emission current, and a working distance (WD) of 8.0 mm.

### 2.5. Evolved Gas Analysis

Coupled TGA-DTA-FTIR analysis of evolved gas during decomposition in a temperature range of 30–900 °C was carried out using STA 449C Jupiter^®^ (Netzsch Gerätebau GmbH, Selb, Germany) coupled to an FTIR spectrometer Tensor 27 (Bruker Corporation, Billerica, MA, United States of America). The gaseous decomposition products were transferred to the FTIR cell with a nitrogen flow of 30 mLmin^−1^ through a Teflon capillary with an inner diameter of 2 mm and a length of 1.5 m. The FTIR cell and transfer capillary were heated to 230 °C.

## 3. Results

### 3.1. Burning Behavior and Char Morphology

Compounds (Table 1) were tested regarding the flammability using UL-94 V test with two different specimen thicknesses of 0.8 mm and 1.6 mm. The UL-94 V results are summarized in Table 2.

As expected non flame retarded polyamide 66 (PA_GF) failed to achieve an UL-94 V classification in both specimen thicknesses. Every specimen showed an afterflame time >60 s and complete decomposition. The compound using solely Zn-DOPOx (**PA_1**) showed poor fire retardancy in the UL-94 V test. Both specimen thicknesses exhibited a total burning up to the sample holder and no pronounced self-extinguishing effect, hence failing the UL-94 V test with long afterflame times. In the case of the sole use of MPP (**PA_2**), a V-2 classification was observed with short afterflame times for the 0.8 mm specimen due to burning melt dripping. Testing of the 1.6 mm series resulted in V-1 classification due to higher burning times after first ignition without any dripping occurring. For the compound using Zn-DOPOx:MPP (**PA_3**), flame retardancy was significantly improved. Both thicknesses achieved V-0 classifications and exhibited the shortest afterflame times of all prepared compounds.

The variation of weight ratios between Zn-DOPOx and MPP (**PA_4** and **PA_5**) led to an increase in afterflame times and resulted in V-2 and n. c. classification of UL-94 V (0.8 mm). Both compounds showed increased afterflame times for the 1.6 mm specimen compared to **PA_3**.

Furthermore, the reduction of the total load of Zn-DOPOx:MPP (1:1) was investigated. With a load of 20% (**PA_6**), the compound failed to achieve an UL-94 V classification of the 0.8 mm specimens due to poor reproducibility within the series. On average, one or two specimens showed burning up to the clamp which disqualifies the whole series. The 1.6 mm specimen was classified as V-0 with slightly longer afterflame times compared to **PA_3**. The 0.8 mm specimen of the 18% variant (**PA_7**) failed as expected in achieving a UL-94 V classification. For the 1.6 mm series, a significant increase in afterflame time was observed while still achieving V-0.

In conclusion, the compound **PA_3** using equal parts of weight between Zn-DOPOx and MPP with a total loading of 22% resulted in the shortest afterflame times and V-0 classifications in UL-94 V. In order to clarify the cause of this improvement, the compounds **PA_1** to **PA_3** were further investigated regarding the mode of action.

**Figure 1:**
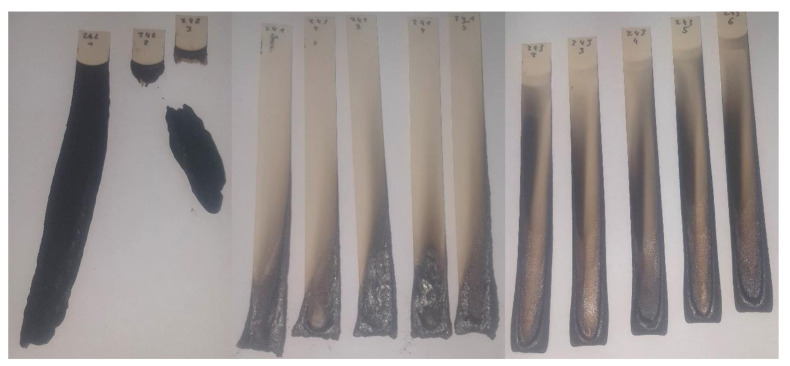
Burnt specimens after UL-94 V testing (1.6 mm). From left to right: **PA_1** to **PA_3**.

In the first step, burnt UL-94 V specimens (1.6 mm) of **PA_1** to **PA_3** were analyzed via SEM-EDX to assess the morphology and elemental composition of the char. Images of the burnt specimens are shown in Figure 1.

**Figure 2:**
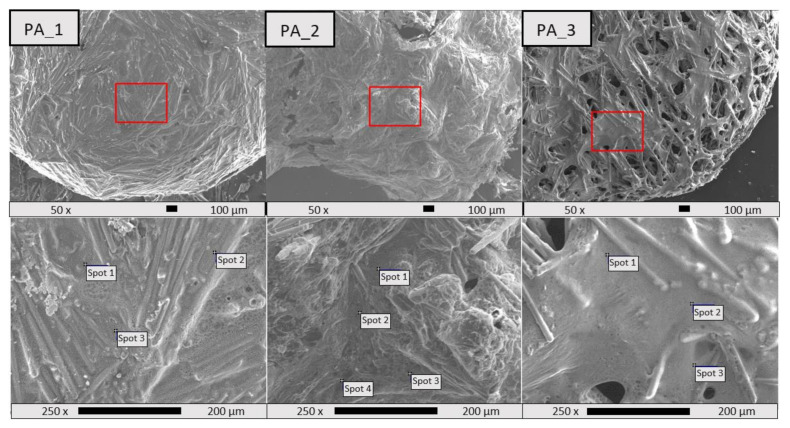
SEM-EDX analysis of a burnt UL-94 V (1.6 mm) specimens of **PA_1** to **PA_3** to investigate the elemental composition of the char. Top: 50× magnification of the area on the lower corner with the investigated area (red square). Bottom: 250× magnification with spots for EDX measurements.

The lower corner of one representative specimen of **PA_1** to **PA_3** was analyzed by SEM-EDX for the elemental compositions. In this area, the difference in char formation was most pronounced because of the highest thermal load due to the high surface area during flaming compared to the edges or flat surface. The resulting surface (50×) and the area used in SEM-EDX analysis (250×) of the char analysis are shown in Figure 2**.**

It can be concluded from the surface images (50×) that the char morphology differs significantly between the compounds. Using Zn-DOPOx (**PA_1**) resulted in an even and a continuously closed layer. In comparison to the other morphologies, it can be stated that the use of Zn-DOPOx (**PA_1**) promoted the formation of a continuous char layer but overall a lack of flame retardancy in UL-94 V was observed. This behavior might indicate a lack of mode of action in the gas phase. Thus, a known gas phase effect for other DOPO(x) derivates was described in the introduction, the effect was not observed for solely Zn-DOPOx in PA 66. This is assigned to the excessive thermal stability of the salt structure compared to the decomposition temperature of PA 66.

For the compound using solely MPP (**PA_2**), a widely closed char layer was observed. In some regions, holes and cracks were visible and due to the release of inert gases, expanded areas were observed. On handling the burnt specimen for the SEM analysis preparation, the char seemed very brittle compared to the other compounds. Because of this, further cracks might have arisen during sample preparation for SEM-EDX. Overall, these observations are in accordance with the well-known ability of MPP to promote char formation [20,23].

In case of the compound using the combination of Zn-DOPOx:MPP (**PA_3**), a high number of holes was visible in the char layer. This change in char morphology indicates that most likely, the decomposition behavior, thermal stability of the char, and overall the mode of action changed significantly versus the individual flame retardants. The open char layer facilitated the release of combustible decomposition products compared to the other morphologies. The short afterflame times and V-0 classification in UL-94 V indicate that the overall effect towards the reduction of flammability was predominant and cannot be assigned to the formation of a more protective layer in the solid phase. Therefore, a stronger gas phase mode of action exists for the use of Zn-DOPOx:MPP (1:1) compared to the single flame retardants.

To evaluate the mechanism further, the elemental composition of the char was determined by SEM-EDX with regard to the relevant elements (C, N, O, P, Zn). In addition, the elemental composition before decomposition was calculated for the elements determined with SEM-EDX using the molar mass and the weight percentages of the components for comparison. In case of Zn-DOPOx, a molar mass of 527.7 gmol^−1^, for MPP (206.1 gmol^-1^) and PA 66 (226.3 gmol^−1^) the molar masses of the repetition units were used. Through the use of the molar mass of the repetition units, the mass of the end groups of the polymeric molecules was neglected. For the analysis of the elemental composition, three spots were measured where no glass fibers were visible. The results of this analysis are shown in Table 3 in atomic percent (at. %) for each element.

The SEM-EDX analysis of the resulting char revealed significant differences in the elemental composition. Regarding the elements C, N, and O, the differences were most pronounced in the oxygen and carbon content. The Zn-DOPOx compound (**PA_1**) showed the highest oxygen content by far and the lowest carbon content. This indicates that the release of carbon-rich fragments was predominant and less oxygen-rich fragments were released during decomposition.

In contrast, the highest carbon and lowest oxygen content was observed for **PA_3** which suggests the pronounced release of CO_2_ and reduced release of carbon rich fragments.

For **PA_2,** the carbon and oxygen content was higher than for **PA_1** but lower than for **PA_3**. As expected, the greatest amount of nitrogen and phosphorus was observed because of the highest melamine content. Moreover, the highest phosphorus content was measured. The phosphorus content remained quantitatively in the solid residue in the form of different oxygen rich phosphorous species such as phosphates, pyrophosphates, and polyphosphates, and increase of oxygen content as described in the literature [20]. It is also reported that the release of CO_2_ is shifted to lower temperatures which leads to the release of oxygen rich decomposition products [23].

The volatile decomposition products were further analyzed to gain more information on the released species.

Regarding the phosphorus content, the lowest values were observed for the compound using the mixture of Zn-DOPOx:MPP (1:1), although for the other two compounds, quite similar values were measured. Overall, this could indicate that the mixture of both flame retardants facilitated the release of phosphorous species into the gas phase in order to act as radical quenching species.

### 3.2. Cone Calorimetry

**Figure 3:**
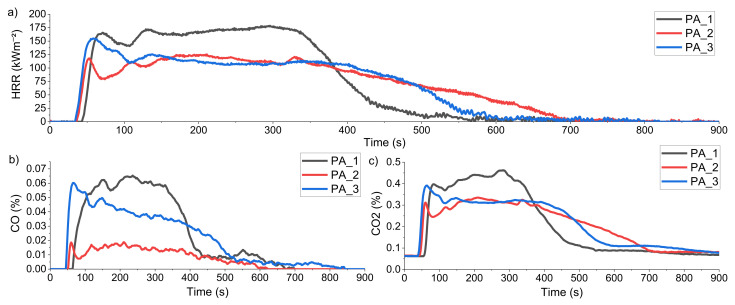
Results of cone calorimetry using an external heat flux of 50 kWm^−2^: (**a**) HRR curves; (**b**) CO-content; and (**c**) CO_2_-content over time for the compounds **PA_1** to **PA_3**.

Cone parameters of **PA_1** to **PA_3** are summarized in Table 4. They include the residue, time to ignition (TTI), burning time, peak of heat release rate (pHRR), total heat release (THR), ratio between THR and total mass loss (TML), and total smoke release (TSR). THR/TML is used for comparison. This quotient indicates the evolving heat per mass which gives an indication for the reduction of THR through gas phase action. Another advantage of this parameter is that it is independent from the sample area. The resulting heat release rate (HRR) curves, carbon monoxide (CO), and carbon dioxide (CO_2_) contents are shown in Figure 3.

**Figure 4:**
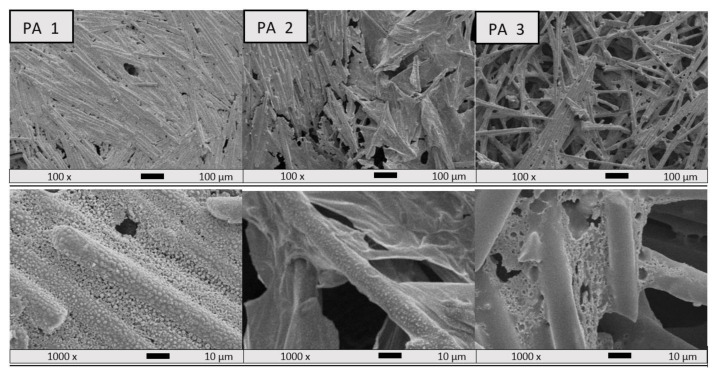
SEM images (top: 100×; bottom: 1000×) of cone residues after testing with an external heat flux of 50 kWm^−2^.

Before the results are discussed, a comment on the total heat release (THR) has to be made because of some experimental observations. Through the occurrence of intumescent behavior after ignition for all compounds, the charred surface of the samples increased in height (1–4 cm). This decrease in distance between the heating element and the sample surface affects the HRR due to the experimental setup of the cone calorimeter and ultimately the THR [46]. This phenomenon can be seen through a second rise in the heat release rate (HRR) curves after the initial peak of heat release rate (pHRR). In addition, some flaming on the edges of the heightened surfaces inside the retainer frame was observed, which also adds up to the THR and yields in similar values for all samples. In order to evaluate the fire behavior of these compounds, the course of the HRR curves has to be discussed predominantly. Additional information regarding the gas phase activity was available through the comparison of the CO content throughout the measurement as shown in Figure 3. A higher CO content may indicate a more pronounced gas phase mechanism by inhibiting total oxidation to CO_2_. Regarding the direct correlation, it has to be stated that on its own, an elevated CO evolution is no clear proof of the radical quenching process because the CO content and smoke production are influenced by several additional factors described in the literature [46]. In order to estimate the effect of pronounced CO evolution, the CO_2_ content was plotted additionally to see if one compound yielded a much higher CO/CO_2_ ratio. Additionally, the protective layer of the cone residues after testing were analyzed by SEM (Figure 4).

Regarding the pHRR, the lowest value was observed for **PA_2** which can be attributed to surface charring before ignition. In case of the other compounds, only bubbles were formed on the molten sample surface before ignition. On ignition, these bubbles burst and released large amounts of combustible decomposition products which ultimately resulted in high pHRR for the compounds **PA_1** and **PA_3**. After ignition, the surface exhibited fast charring behavior after about 8 s to 13 s.

Ignition of **PA_1** occurred later compared to the other compounds. After about 120 s, pHHR was increased through foaming which resulted in a height increase of the sample surface of about 2–3 cm. At about 350 s, the HRR decreased significantly and resulted in the earliest flameout of all compounds. After this short burning time, the whole sample was charred and no neat polymer underneath was observed. In comparison to **PA_2** and **PA_3,** this compound led to the highest smoke and CO and CO_2_ evolution. The surface of the residue consisted of a nearly continuously closed char after cone calorimetry which is like that as observed after UL-94 V testing.

The sole use of MPP (**PA_2**) resulted in a second rise in HRR due to intumescent behavior. In total, the increase in height was more pronounced with 3–4 cm than for **PA_1**. In addition, the HRR values were significantly lower with approximately 110–120 kWm^−2^ after the first maximum is reached. For **PA_2,** the lowest smoke production and CO evolution were observed. Regarding the CO_2_ evolution, similar values to PA_3 were obtained between 150 s and 500 s but **PA_2** also showed the longest evolution, continuing up to approximately 700 s. Through effective barrier formation (Figure 4) and, therefore, slower release of fuel, the longest burning time was observed. Like burnt UL-94 V specimens, the surface showed more cracks and holes than **PA_1**. These observations are in accordance to the reported mode of action in the literature through dilution, barrier formation, and lack of fuel poisoning [20,23].

An effective protective layer was formed of **PA_3** after ignition at around 150 s and led to the decrease in HRR after the first maximum was reached to the level of around 100–120 kW m^−2^, which is comparable to the HRR values of **PA_2**. At around 400 s, the HRR started to decrease more rapidly for **PA_3** than it did for **PA_2**. This indicates the formation of a less stable protective layer which was confirmed through SEM analysis of the specimen after testing. The height increase of around 2–3 cm for **PA_3** was comparable to **PA_1**. Despite the absence of a protective layer (Figure 4), the relatively low HRR and V-0 classification in UL-94 V showed an overall increase in flame retardancy, which was assumed to be achieved by a stronger gas phase activity. Furthermore, for **PA_3** the lowest value of THR/TML was observed, meaning that the least amount of heat was produced per mass which supports the suggested mode of action in the gas phase. By comparing the HRR curves and CO evolution of **PA_2** and **PA_3,** this conclusion is obvious. Despite similar HRR values between 130 s and 500 s, the CO evolution of **PA_3** exceeded the value of **PA_2** by three times. The high CO/CO_2_ ratio of **PA_3** and high smoke production was an indication for a mechanism through fuel poisoning in the gas phase which seemed to compensate for the less pronounced solid phase activity caused by the open framework structure of the char [46]. In order to prove the release of P-species and ultimately the radical quenching effect, P-species have to be identified through additional analysis, such as mass spectrometry [17].

### 3.3. Thermogravimetric Analysis of Flame Retardants

Thermogravimetric analysis was used to investigate the interaction between Zn-DOPOx and MPP in the mixature using equal parts of weight. In order to assess a change through interaction in the expected mass ratio of the residue (*R_calc_*), the value for the mixture (1:1) was calculated by considering the weight percentage (*w_i_*) and using the mass ratio values of the TGA data of Zn-DOPOx (*R_Zn-DOPOx_*) and MPP (*R_MPP_*) according to Equation (1).

(1) $$R_{calc}=\frac{w_{Zn-DOPOx}R_{Zn-DOPOx}+w_{MPP}\;R_{MPP}}{w_{Zn-DOPOx}+w_{MPP}}\;and\;w_{Zn-DOPOx}=w_{MPP}=0.5$$

**Figure 5:**
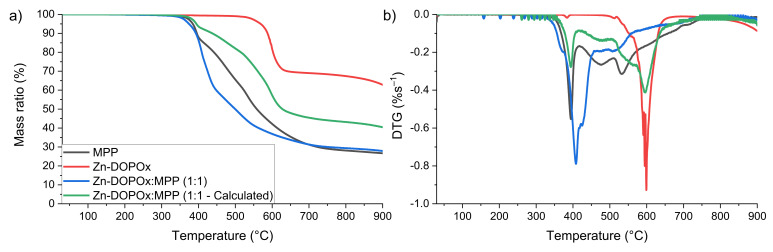
(**a**) Thermogram and (**b**) DTG of the experimental and calculated values under nitrogen in a temperature range between 30 °C and 900 °C.

The comparison of all thermograms and derivative thermograms (DTG) are shown in Figure 5 and the thermogravimetric data are summarized in Table 5.

As known from the literature [38], Zn-DOPOx exhibits high thermal stability. The main decomposition step took place in a temperature range of 550–650 °C, after which only a slight mass loss is observed resulting in a mass ratio of 54% at 900 °C. The onset temperature for MPP was in the range of 330 °C and showed a rapid mass loss between 330 °C and 420 °C after which the mass loss was almost linear until approximately 650 °C. Above this temperature, only slow mass loss occurred and a mass ratio of 26% was observed at 900 °C. When the calculated and experimental mass ratio for Zn-DOPOx:MPP (1:1) were compared, a significant change in decomposition behavior was evident which indicates a strong interaction between the components. As expected, the onset temperature was in the range of MPP. After the onset, the mass loss rate exceeded the others during the main decomposition step (370–550 °C) and the mass loss ascribed to Zn-DOPOx decomposition was not visible in the DTG, which is another indication for the significant change in thermal stability. Additionally, the experimentally found mass ratio from Zn-DOPOx:MPP (1:1) was significantly lower than expected based on the calculated value from the individual TGA curves. Surprisingly, the mass ratio was very similar to the values for MPP above 700 °C and resulted in 28% instead of the calculated 40% at 900 °C.

**Figure 6:**
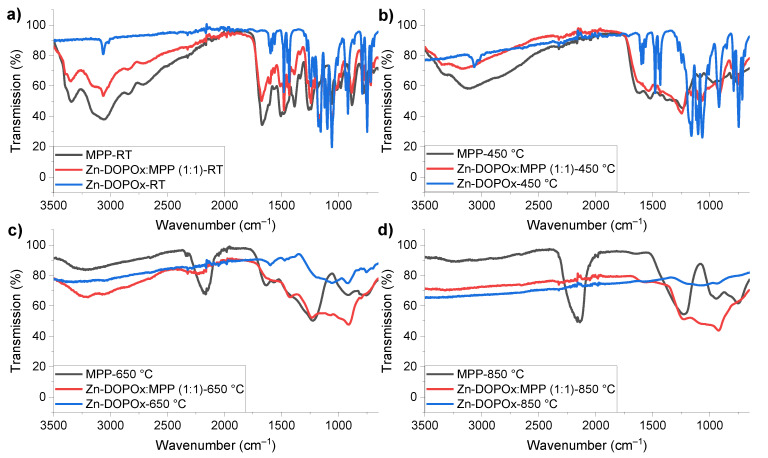
ATR-FTIR spectra of (**a**) neat Zn-DOPOx, MPP, Zn-DOPOx:MPP (1:1), and TGA residues prepared under nitrogen at (**b**) 450 °C; (**c**) 650 °C; and (**d**) 850 °C.

Finally, TGA residues taken from different temperatures (450, 650, 850 °C) under inert conditions were analyzed using ATR-FTIR towards characteristic changes in IR absorption peaks. The spectra from the residues and the initial spectra before decomposition (RT = room temperature) for Zn-DOPOx, MPP, and the binary combination using equal parts of weight (Zn-DOPOx:MPP (1:1)) are shown in Figure 6.

The thermogram of Zn-DOPOx exhibits the main decomposition step in the temperature range 550–650 °C. Above 650 °C, a molten and foamed black residue was obtained. At 650°C, the peaks caused by the aromatic C−H vibrations (approx. 3000 cm^−1^) and C−H (approx. 1500 cm^−1^) decreased significantly. After the main decomposition, no sharp peaks were observed and only a very broad absorption pattern with maxima at 1190, 1050, 916, and 754 cm^−1^ was measured. In this region, characteristic phosphorous vibrations such as P−OC_arom._ (1240 cm^−1^), P−C_arom._ (1150 cm^−1^), and P−O−P (980 cm^−1^) were found [47]. Up to 750 °C, only slight changes could be observed but overall, the intensity of the absorption was very low. The broad absorption pattern of the residue in the fingerprint region indicates an amorphous residue [48], probably consisting of a complex mixture of different phosphorous species and undefined organic residues.

For MPP, the main decomposition step occurred in the temperature range of 350–650 °C. Up to 750 °C, the residues were obtained as a white powder whereas the color changed to slightly yellow above this temperature. When comparing the initial ATR-FTIR spectra with the spectra obtained at higher temperatures, a significant decrease in N−H (3400–3100 cm^−1^) and C=N (1690–1410 cm^−1^) absorption bands was visible. At 650 °C, only small amounts of N−H absorption bands could be seen which disappeared completely at 750 °C. In addition, two major changes in the spectra of MPP at higher temperatures were detected. Firstly, the formation of the very pronounced peak at 2160 cm^−1^ was observed and secondly, the changes in the fingerprint region (<1500 cm^−1^) were detected. The absorption band at 2160 cm^−1^ was located in the range of cumulative double bonds such as carbodiimides (−C=N=C−) or nitriles (−C≡N) [47]. In the fingerprint region, characteristic phosphorous absorption peaks from P=O (1260 cm^−1^) and P−O−P (980, 760 cm^−1^) were obtained up to higher temperatures whereas C- and N-based absorption bands decreased. In general, a change to broad peaks in comparison to the initial spectra before heat treatment could be seen in all samples. It can be assigned again to a complex mixture of different phosphorous species and undefined organic residues.

The spectrum of Zn-DOPOx:MPP (1:1) resulted in a major change in the absorption pattern as concluded from the absence of the pronounced absorption band formed during MPP decomposition at 2160 cm^−1^. The decrease in N−H absorption bands (3400–3100 cm^−1^) and triazine peaks (1690–1410 cm^−1^ and 780 cm^−1^) could be seen up to a temperature of 750 °C where no triazine absorptions were left. Sharp peaks caused by the aromatic C−H bonds of the DOPOx molecule (3060 cm^−1^) were visible in the initial spectrum of the mixture but disappeared in the spectra at 450 °C contrary to Zn-DOPOx.

It can be concluded that the ATR-FTIR spectra confirmed a change in decomposition for Zn-DOPOx:MPP (1:1) compared to the single flame retardants as discussed for the triazine and DOPOx decomposition. At 750 °C, the spectrum of the residue showed a high similarity to the spectra of different zinc phosphates which were investigated in a study regarding the structure of phosphate glasses [49]. When comparing the relative intensities of the resulting peaks and the fingerprint area to those of different zinc phosphates such as ortho-, pyro-, meta-, and polyphosphates [49], a high similarity to the ortho- and pyrophosphate spectra are obvious. Therefore, it is concluded that the residue of Zn-DOPOx:MPP (1:1) consists mainly of zinc phosphates with additional undefined charred organic residue. The formation of these species is described in the literature for other metal phosphorus salts, such as DEPZn [50] or melamine-poly(zinc phosphate) [25]. In order to identify the formed species, further analysis such as ^31^P-MAS NMR [23,49] is necessary to confirm the hypothesis regarding the formation of zinc phosphates through ion exchange between Zn-DOPOx and MPP.

### 3.4. Thermal and Evolved Gas Analysis of Flame Retarded Compounds

Differential thermal analysis (DTA) analysis allows to identify exo- or endothermic decomposition behavior. Simultaneous analysis of gaseous decomposition products by using FTIR spectroscopy reveals the release of different volatile fragments formed by PA 66. Schartel et al. [23] showed that the observation of a few key decomposition products is sufficient for the assessment regarding flammability. These are the inert decomposition products such as NH_3_, CO, CO_2_, and H_2_O, as well as combustible fragments such as aliphatic amines and hydrocarbons such as cyclopentanone.

**Figure 7:**
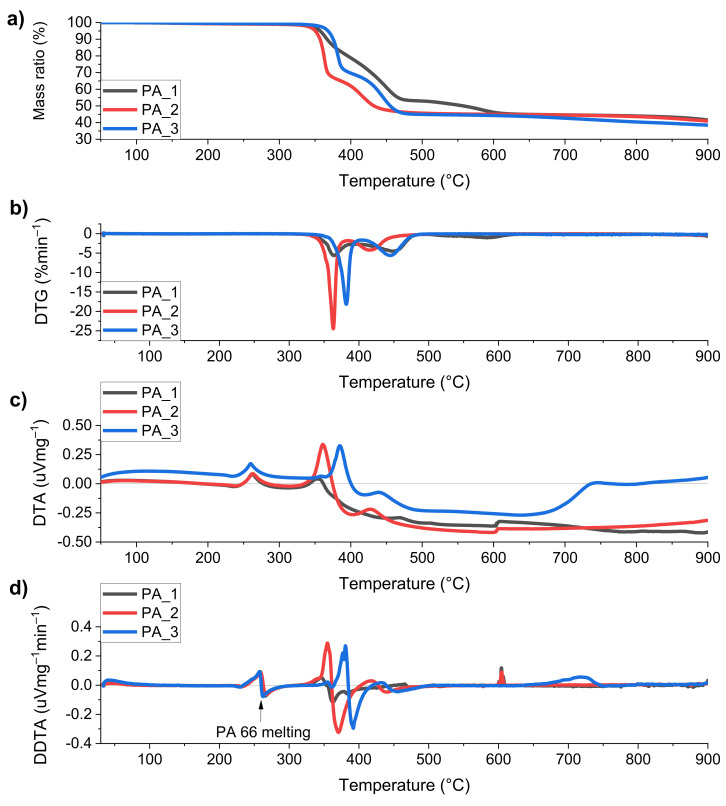
Results of the DTA analysis of **PA_1** to **PA_3** in the temperature range 30–900 °C under nitrogen: (**a**) thermogram; (**b**) DTG; and (**c**) DTA; (**d**) DDTA curves regarding endo- and exothermic decomposition.

For the evaluation of the thermal behavior, the derivative of the DTA curve (DDTA) had to be taken into consideration because the sample holder was not fixed in this experimental setup. This led to a shift in the DTA values on mass loss which distorted the interpretation of the endo- and exothermic nature of the ongoing processes. Through the DDTA curve, the processes could be evaluated based on their gradient pattern during a DTA change. An endothermic process could be assigned through the DDTA curve exhibiting a wave-like pattern with first a rise to positive values and afterwards changing to negative values. After the process, the DDTA values returns to nearly zero and the distortion in the baseline signal of DTA is corrected. An exothermic process shows the opposite wave pattern in the DDTA signal. In order to evaluate the flame retarded formulations, the thermograms, DTG, DTA, and DDTA curves for **PA_1** to **PA_3** are shown in Figure 7. In addition, the most important parameters regarding the thermal stability are summarized in Table 6. Through the DTGmax value, the temperature at maximum decomposition was obtained, which is an important factor in a fire scenario. The values of non-flame retarded PA 66 GF (measured as described in Section 2.2) are also shown for comparison of the charring induced by the flame retardants.

The first endothermic pattern visible in DDTA can be assigned to the melting of PA 66 (240–280 °C). The high overlap of all DDTA patterns between **PA_1** and **PA_3** shows that the crystallization was not significantly changed through the flame retardant systems while having the same PA 66 content. For **PA_1**, the onset of decomposition was observed at 340 °C followed by the slowest thermal decomposition and resulting in the highest thermal stability of all compounds up to approximately 620 °C. Two decomposition steps were visible for **PA_1**. During the first step, a more rapid decrease and a greater mass loss between 340 °C and 500 °C was detected while the second step only exhibited a small mass loss between 500 °C and 650 °C. Comparing the DTG values of **PA_1** to the others, it was obvious that the decomposition took place much slower while having a maximum at 364 °C. The second step can be assigned to Zn-DOPOx decomposition through the correlation with the thermogram (Figure 5). The mass ratio at 900 °C was similar to **PA_2** and correlated to the higher stability of the protective layers of **PA_1** and **PA_2**. DDTA analysis revealed that only one minimum endothermic pattern (340–360 °C) existed after initial decomposition for **PA_1** in contrast to **PA_2** and **PA_3**. Above 360 °C, no pronounced endo- or exothermic decomposition was observed for **PA_1**. This shows that the lack of a pronounced endothermic pattern during the early stage of decomposition indicated that no heat sink mechanism exists and facilitates the decomposition through thermal feedback and thus the release of fuel.

The onset for **PA_2** (330 °C) and the observed two step decomposition behavior was in accordance with the expectations from the literature [23]. Due to the higher flame retardant loading in the current study compared to the investigations by Schartel et al. [23], the observed mass ratio was higher with 40.9% at 900 °C. Compared to **PA_1** and **PA_3**, the lowest thermal stability was observed with the lowest mass ratio between 380 °C and 470 °C. In the range between 470 °C and 670 °C, the mass ratio was nearly identical with **PA_3** but the latter showed an additional decrease up to 900 °C. For **PA_2**, the maximum rate of decomposition was observed at 364 °C. In contrast to **PA_1**, a pronounced endothermic DDTA pattern in the early stage of thermal decomposition was detected in the range between 330 °C and 400 °C which is favorable for a flame scenario by acting as a heat sink in the condensed phase in an early stage of the decomposition. Between 400 °C and 470 °C, only a small endothermic DDTA pattern was observed which expanded the temperature range of the heat sink mechanism. For **PA_1** and **PA_2**, a very sharp DDTA signal at 600 °C was measured. This is ascribed to an experimental error and was not further used for the evaluation of the compounds.

**PA_3** showed the highest onset of decomposition at about 350 °C. The course of the two-step decomposition behavior (1st step at 350–410 °C; 2nd step at 410–480 °C) was quite similar to **PA_2** but is shifted towards higher temperatures. Especially, the temperature of maximum decomposition was shifted to 381 °C. For **PA_3**, an additional small mass loss above 650 °C was observed, resulting in the lowest mass ratio in comparison to **PA_1** and **PA_2**. This explains the lack of a protective layer in the burnt specimen. Similar to **PA_2**, a pronounced endothermic DDTA pattern was obtained but shifted towards 350–410 °C. Between 410 °C and 490 °C, an additional small endothermic DDTA pattern was observed, similar to **PA_2**. Overall, **PA_3** showed the broadest temperature range (350–490 °C) of early endothermic decomposition which acts as a heat sink up to higher temperatures. Surprisingly, the additional mass loss (>650 °C) resulted in a small endothermic DDTA signal between 650 °C and 740 °C, which can act also as a heat sink at higher temperatures. Overall, **PA_3** exhibited two favorable factors in decomposition regarding a fire scenario. Firstly, the pronounced endothermic peak through the use of MPP acted as a heat sink during char formation. Secondly, the broad temperature range of endothermic and additional small endothermic contribution at higher temperatures were favorable for a decrease in decomposition and therefore, fuel release. Yet the additional mass loss was responsible for the observed open char structure of the burnt specimen due to the lowest mass ratio.

**Figure 8:**
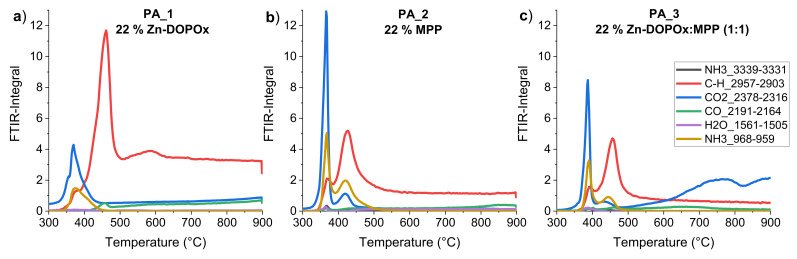
FTIR integrals of volatile PA 66 decomposition products over the decomposition between 300 °C and 900 °C under nitrogen for (**a**) **PA_1**; (**b**) **PA_2**; and (**c**) **PA_3**.

The detection of PA 66 decomposition products was performed by assigning characteristic FTIR vibrations according to Table 7. It has to be mentioned that due to possible condensation in the transfer capillary, not all resulting decomposition products were detected using this method, especially ammonia or substituted derivates of cyclopentanone are described as prone to condensation in the literature [23]. Figure 8 shows the release of the volatile decomposition products under nitrogen atmosphere after decomposition started (>300 °C). The wavenumber range used for integration and the temperatures of the maximum release for each characteristic FTIR vibration are summarized in Table 7.

In order to estimate the effect of fuel dilution caused by the release of inert gases (CO_2_ and NH_3_), the ratios of the maxima between the two inert gases and C−H fragments were compared.

**PA_1** showed the highest release of combustible C−H fragments and the lowest integrals for inert gases such as CO_2_ and NH_3_ of all compounds. The release of CO_2_ and NH_3_ was observed in the temperature range of 330–440 °C with a maximum at approx. 370 °C. In the same range, the release of C−H fragments was detected with a shoulder at 370 °C. Above this temperature, the release showed a significant increase and exhibited the highest values of all compounds. Above the maximum at 461 °C, the release decreased but continued up to 900 °C. The release of CO was only detected to a minor extent with a maximum at 460 °C. During the release of C−H fragments at higher temperatures, very small amounts of CO and CO_2_ were released simultaneously which yielded a flammable mix of decomposition products.

The release of gaseous decomposition products for **PA_2** was in accordance with the behavior described in the literature [23]. After initial decomposition, the highest release of CO_2_ of all the compounds **PA_1** to **PA_3** was observed. In the early stage of decomposition, the release of CO_2_, NH_3_, and C−H fragments was observed. The temperature of decomposition shifted towards lower temperatures for **PA_2** (330–390 °C). In this temperature range, the ratio of inert gases to C−H vibrations roughly doubled compared to **PA_1**. The main release of C−H fragments was observed between 390 °C and 480 °C with a noticeably lower integral compared to **PA_1**. A significant increase in the simultaneous release of CO_2_ and especially NH_3_ was detected for **PA_2**. These observations prove the known gas phase effect of MPP through dilution. At higher temperatures (>500 °C), the C−H vibration integral indicated a continuous slow release up to 900 °C. The production of CO and H_2_O was only slightly visible during the first decomposition step. For CO, a minimal release at temperatures above 700 °C was observed.

**PA_3** showed the first stage of decomposition (350–410 °C) with mainly the release of CO_2_ and NH_3_ and C−H fragments. Compared to **PA_2**, the CO_2_ integral was noticeably lower but due to the simultaneous decrease in C−H vibrations, the ratio of inert gases to C−H (Table 7) was in a similar range. Compared to **PA_1**, a significant higher content of inert gases was observed. This ratio suggests an effect through fuel dilution for the mixture Zn-DOPOx:MPP (1:1). During the main release of C−H fragments (410–520 °C), a similar maximum integral value as for **PA_2** was detected but overall lower amounts of inert fragments. The release of C−H fragments dropped to lower rates at higher temperatures (>500 °C) than for **PA_1** and **PA_2**. Simultaneously, **PA_3** exhibited an additional CO_2_ release above 480 °C which may act as a continuous dilution probably due to the oxidation of the less stable char which is responsible for the open char structure observed by SEM imaging. The continuous release of CO_2_ up to higher temperatures also explains the low oxygen content detected in the SEM-EDX analysis of burnt UL-94 V specimens. Overall, a very low release of CO was detected under inert DTA-FTIR conditions. The lack of high amounts of CO for **PA_3** supports the thesis that flame poisoning through the release of phosphorous species induced the high CO content observed during cone calorimetry, which is in line with the low phosphorus content in SEM-EDX.

In conclusion, two beneficial factors of the decomposition behavior were found. Through the significant reduction in exothermic behavior, the internal contribution to thermal feedback was reduced and the pronounced endothermic peak led to a deceleration in the initial early-stage decomposition. Additionally, the mixture of volatile decomposition products exhibited a high portion of inert gases (CO_2_, NH_3_), similar to **PA_2**, and fewer C−H fragments than **PA_1** and **PA_2**. In combination, a mode of action in the condensed phase through less internal thermal feedback and a mode of action in the gas phase through fuel dilution and probably flame poisoning were identified.

## 4. Conclusions

Zn-DOPOx, MPP and especially their mixture as flame retardants in PA 66GF were investigated towards the mode of action. For Zn-DOPOx:MPP (1:1) (**PA_3**), very short afterflame times and V-0 classification (0.8 mm) with a total loading of 22% were observed. The synergistic effect for Zn-DOPOx:MPP (1:1) became obvious when comparing the higher afterflame times and burning melt dripping (0.8 mm) for solely MPP (**PA_2**) and the very poor fire extinguishing effect for using solely Zn-DOPOx (**PA_1**) in the UL-94 V test. The reason for the latter was found to be the excessive thermal stability of Zn-DOPOx to act efficiently in PA 66 as well as an overall release of high amounts of combustible C−H fragments and no pronounced heat sink in the condensed phase. Nevertheless, a char-promoting effect for Zn-DOPOx (**PA_1**) could be seen using SEM. For solely MPP (**PA_2**), a mode of action through fuel dilution and barrier formation was observed.

Based on a 22% loading, the ratio variation led to the best flame retarding effect for equal parts of weight in contrast to Zn-DOPOx:MPP = 2:1 (**PA_4**) or 1:2 (**PA_5**). TGA and ATR-FTIR analyses revealed a strong interaction between the components through a significant change in thermal stability and in absorption patterns of TGA residues for Zn-DOPOx:MPP (1:1). A major change in decomposition was also observed in PA 66 GF (**PA_3)** compared to the sole use of Zn-DOPOx (**PA_1**) and MPP (**PA_2**) using DTA-FTIR analysis. It was shown for **PA_3** that the intrinsic contribution to the thermal feedback through exothermic decomposition was reduced significantly and fewer C−H fragments were detected compared to **PA_1** and **PA_2**. For **PA_3**, a high portion of inert gases was released upon decomposition which diluted the fuel in the gas phase while the pronounced endothermic DDTA pattern (350–490 °C) revealed the mode of action through heat sink in the condensed phase. The latter can be attributed to the use of MPP as seen in **PA_2**. By using cone calorimetry, it was shown that despite the open char structure of **PA_3**, low HRR was achieved similar to **PA_2**. In combination with the significantly higher CO/CO_2_ ratio and smoke production of **PA_3**, it was concluded that this was an indicator for a possible mode of action through radical quenching through the interaction of the components in Zn-DOPOx:MPP (1:1). This is supported by the lowest phosphorus content in burnt UL-94 V specimen measured with SEM-EDX. Because the CO content and smoke production were influenced by additional factors, a validation of the release of P-species has to be made through additional analysis which is a possible key point for future research. Overall, the reduced flammability through the observed gas phase action and the beneficial changes in thermal decomposition regarding the pronounced mode of action through heat sink and release of less C−H fragments with high portion of inert gases led to the improvement in flame retardancy of the mixture Zn-DOPOx:MPP (1:1). An interesting topic for further studies would be the improvement of char stability and morphology while using the observed improved gas phase activity for the combination of Zn-DOPOx:MPP (1:1).

## Figures and Tables

**Table 1:** Overview of flame retardant compositions used in the PA 66 GF compounds based on Zn-DOPOx and MPP.

Flame Retardants (wt. %)	Compound						
	PA_1	PA_2	PA_3	PA_4	PA_5	PA_6	PA_7
**Zn-DOPOx**	22	–	11	14.7	7.3	10	9
**MPP**	–	22	11	7.3	14.7	10	9

**Table 2:** Results of UL-94 V testing of formulations using Zn-DOPOx and MPP in PA 66 GF.

Compound	Flame Retardant		UL-94 V (0.8 mm)		UL-94 V (1.6 mm)	
	Zn-DOPOx (wt. %)	MPP (wt. %)	Total Afterflame (s)	Classification	Total Afterflame (s)	Classification
**PA_GF**	–	–	>300	n.c.	>300	n.c.
**PA_1**	22	–	80.6	n.c.	182.3	n.c.
**PA_2**	–	22	15.6	V-2	52.7	V-1
**PA_3**	11	11	10.4	V-0	4.7	V-0
**PA_4**	14.7	7.3	38.6	V-2	5.5	V-0
**PA_5**	7.3	14.7	56.8	n.c.	7.9	V-0
**PA_6**	10	10	11.3	n.c.	6.7	V-0
**PA_7**	9	9	93.7	n.c.	42.3	V-0

**Table 3:** SEM-EDX results of spots in the burnt area on the lower edge of UL-94 V (1.6 mm) specimen.

Element	PA_1 22 % Zn-DOPOx		PA_2 22 % MPP		PA_3 22 % Zn-DOPOx:MPP (1:1)	
	Calculated (at. %)	SEM-EDX (at. %)	Calculated (at. %)	SEM-EDX (at. %)	Calculated (at. %)	SEM-EDX (at. %)
**C**	74.3	44.5 ± 6.5	59.9	53.1 ± 6.9	67.1	76.1 ± 6.1
**N**	8.9	8.7 ± 3.6	22.3	10.5 ± 1.9	15.6	8.5 ± 1.4
**O**	14.1	33.1 ± 4.6	15.6	20.8 ± 3.4	14.9	8.0 ± 3.9
**P**	1.8	7.8 ± 3.4	2.2	7.9 ± 1.1	2.0	5.3 ± 0.3
**Zn**	0.9	3.7 ± 1.6	0	–	0.4	1.6 ± 0.5

**Table 4:** Results of the cone calorimetry for the compounds **PA_1** to **PA_3** with an external heat flux of 50 kWm^−2^.

Compound		PA_1 22% Zn-DOPOx	PA_2 22% MPP	PA_3 22% Zn-DOPOx:MPP (1:1)
Residue	(%)	36.7 ± 0.7	41.4 ± 1.5	35.1 ± 0.4
TTI	(s)	44 ± 4	35 ± 1	31 ± 4
Burning time	(s)	537 ± 17	694 ± 13	661 ± 26
pHRR	(kWm^−2^)	188.9 ± 10.7	123.0 ± 5.3	162.5 ± 6.9
THR	(MJm^−2^)	57.8 ± 1.7	54.1 ± 1.8	57.1 ± 3.4
THR/TML	(kWm^−2^g^−1^)	2.02 ± 0.03	2.00 ± 0.08	1.91 ± 0.08
TSR	(m^2^)	1619 ± 73	502 ± 60	1498 ± 97

**Table 5:** Thermogravimetric data of Zn-DOPOx, MPP and Zn-DOPOx (1:1–experimental and calculated).

Sample	T_95_ (°C)	T_90_ (°C)	DTG_max_ (°C)	Mass Ratio (600 °C) (%)	Mass Ratio (900 °C) (%)
Zn-DOPOx	543	563	600	72.5	54.2
MPP	385	396	399	42.2	26.2
Zn-DOPOx:MPP (1:1)	374	393	408	36.9	27.9
Zn-DOPOx:MPP (1:1–Calculated)	394	437	598	57.3	40.4

**Table 6:** Results of thermal stability of **PA_1** to **PA_3** and non-flame retarded PA 66 GF using DTA.

Compound	T_95_ (°C)	T_90_ (°C)	DTG_max_ (°C)	Mass Ratio (600 °C) (%)	Mass Ratio (900 °C) (%)
**PA 66 GF** **(without flame retardant)**	390	399	426	32.0	31.2
**PA_1** **22% Zn-DOPOx**	357	367	364	46.2	41.5
**PA_2** **22% MPP**	350	357	363	45.0	40.9
**PA_3** **22% Zn-DOPOx:MPP (1:1)**	368	375	381	44.2	38.5

**Table 7:** DTA-FTIR results for PA 66 decomposition products for **PA_1** to **PA_3** under nitrogen.

Species	Vibration	Wavenumber Range Used for Integration (cm^−1^)	PA_1	PA_2	PA_3
			Peak (°C)	Peak (°C)	Peak (°C)
Amine/NH_3_	N−H VS	3339–3331	375	368	456
C−H	C−H VS	2957–2903	461	426	456
CO_2_	C=O VS	2378–2316	370	366	387
CO	C=O VS	2191–2164	>895	848	663
H_2_O	O−H VS	1719–1651	–	362	387
NH_3_	N−H DS	968–959	375	368	391
I(CO_2_):I(NH_3_):I(C−H) at first maximum (approx. 370 °C) and normalized to I(C−H)			3.4:1.2:1	6.2:2.4:1	5.4:2.1:1

## Data Availability

The data presented in this study are available on request from the corresponding author.
